# Emerging roles of BMP9 and BMP10 in hereditary hemorrhagic telangiectasia

**DOI:** 10.3389/fgene.2014.00456

**Published:** 2015-01-08

**Authors:** Emmanuelle Tillet, Sabine Bailly

**Affiliations:** ^1^Inserm, U1036, Grenoble, France; ^2^Laboratoire Biologie du Cancer et de l’Infection, Institut de Recherches en Technologies et Sciences pour le Vivant, Commissariat à l’énergie atomique et aux énergies alternatives, Grenoble, France; ^3^Université Grenoble-Alpes, Grenoble, France

**Keywords:** hereditary hemorrhagic telangiectasia, BMP9, BMP10, ALK1, endoglin, cell signaling

## Abstract

Rendu–Osler–Weber syndrome, also known as hereditary hemorrhagic telangiectasia (HHT), is an autosomal dominant vascular disorder. Three genes are causally related to HHT: the *ENG* gene encoding endoglin, a co-receptor of the TGFβ family (HHT1), the *ACVRL1* gene encoding ALK1 (activin receptor-like kinase 1), a type I receptor of the TGFβ family (HHT2), and the *SMAD4* gene, encoding a transcription factor critical for this signaling pathway. Bone morphogenetic proteins (BMPs) are growth factors of the TGFβ family. Among them, BMP9 and BMP10 have been shown to bind directly with high affinity to ALK1 and endoglin, and BMP9 mutations have recently been linked to a vascular anomaly syndrome that has phenotypic overlap with HHT. BMP9 and BMP10 are both circulating cytokines in blood, and the current working model is that BMP9 and BMP10 maintain a quiescent endothelial state that is dependent on the level of ALK1/endoglin activation in endothelial cells. In accordance with this model, to explain the etiology of HHT we hypothesize that a deficient BMP9/BMP10/ALK1/endoglin pathway may lead to re-activation of angiogenesis or a greater sensitivity to an angiogenic stimulus. Resulting endothelial hyperproliferation and hypermigration may lead to vasodilatation and generation of an arteriovenous malformation (AVM). HHT would thus result from a defect in the angiogenic balance. This review will focus on the emerging role played by BMP9 and BMP10 in the development of this disease and the therapeutic approaches that this opens.

## HHT IS LINKED TO THE TGFβ SUPERFAMILY SIGNALING PATHWAYS

Three genes are causally related to hereditary hemorrhagic telangiectasia (HHT): the *ENG* gene encoding the co-receptor endoglin (HHT1; McAllister et al., 1994), the *ACVRL1* gene encoding ALK1 (Johnson et al., 1996), and the *SMAD4* gene, a critical factor in this signaling pathway (Gallione et al., 2004). HHT disease is thus clearly linked to the TGFβ superfamily signaling pathways.

**FIGURE 1:**
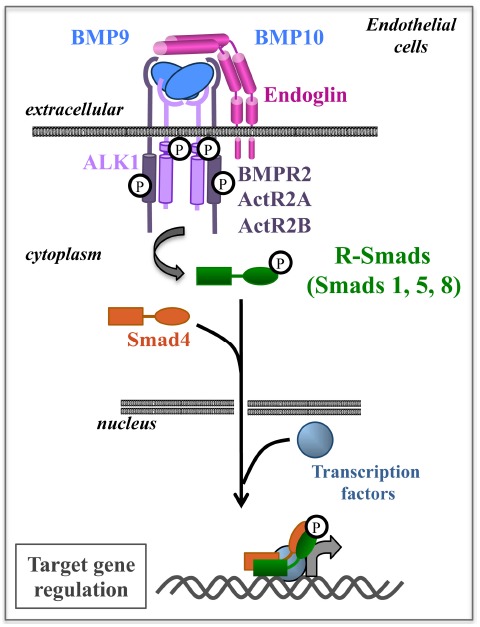
**BMP9/10 are ligands of the ALK1-Endoglin receptor complex and activate the Smad pathway in endothelial cells.** BMP9/10 bind to a heterotetrameric complex composed of two type I receptors (ALK1) and two type II receptors (BMPR2, ActR2A, or ActR2B). Endoglin is a co-receptor of this complex and enhances signaling. Following ligand binding, receptors are phosphorylated and propagate signal through R-Smad 1, 5, 8 phosphorylation. The R-Smads then associate with Smad4 to regulate target gene transcription in the nucleus.

In TGFβ superfamily signaling, ligands bind a heterotetrameric complex composed of two type II receptors and two type I receptors, both of which are serine/threonine kinases. Upon ligand binding, the type II receptors phosphorylate and activate the type I receptor. The activated type I receptor then propagates the signal by phosphorylating a family of transcription factors, the Receptor regulated-Smads (R-Smads). The phosphorylated R-Smad complex, with the common partner Smad4, enters the nucleus and, together with other transcription factors, regulates transcription of target genes (Massague, 2008; Xu et al., 2012; Figure 1). The Smad signaling pathway is the canonical signaling pathway for the TGFβ superfamily; however, non-Smad signaling pathways are also important (Guzman et al., 2012; Poorgholi Belverdi et al., 2012).

There are seven type I (also known as activin receptor-like kinase, i.e., ALK1–7), and five type II receptors. These receptors can also associate with type III receptors, also termed co-receptors, such as endoglin, which increase ligand signaling but have no intrinsic enzymatic activity (Meurer et al., 2014). All these receptor complexes bind to a large family of ligands (33 members in mammals) defining two different pathways: the TGFβ/activin subfamily activates R-Smad2 and 3 while the bone morphogenetic protein (BMP) subfamily activates R-Smad1, 5, and 8 (Figure 1). Many members of this pathway are involved in vascular development and vascular diseases (ten Dijke and Arthur, 2007; Dyer et al., 2014), but this review will focus on receptors and signaling related to HHT pathology.

ALK1 and endoglin belong to a receptor complex that is specifically expressed in endothelial cells and mutations in these receptors are therefore related to vascular defects. This has been confirmed by murine models. In mice, inactivation of either *Alk1* or *Eng* is lethal at mid-gestation, with severe vascular disorders including arteriovenous shunts, vascular dilation, and irregular vascular smooth muscle cell recruitment (Bourdeau et al., 1999, 2000; Li et al., 1999; Arthur et al., 2000; Oh et al., 2000; Urness et al., 2000). Mice heterozygous for *Alk1* or *Eng*, or conditionally deleted for either of these genes in endothelial cells, represent animal models for HHT (Srinivasan et al., 2003; Torsney et al., 2003; Park et al., 2009; Mahmoud et al., 2010).

ALK1 has long been an orphan receptor while the coreceptor endoglin was shown to bind many members of the TGFβ superfamily (Barbara et al., 1999). In 2002, TGFβ1 and TGFβ3 isoforms were first proposed as ligands for ALK1 and endoglin together in a complex with ALK5, the canonical receptor for TGFβ1 and TGFβ3 (Goumans et al., 2002). However, in 2007, BMP9 and BMP10 were found to bind ALK1 and endoglin with a much higher affinity (Brown et al., 2005; David et al., 2007; Scharpfenecker et al., 2007) and recent data further support the notion that BMP9 and BMP10 are the two major ligands of ALK1 (Ricard et al., 2010; Nolan-Stevaux et al., 2012; Park et al., 2012; van Meeteren et al., 2012). In the present manuscript, we will discuss why we think that BMP9 and BMP10 are the most probable ligands for ALK1 and endoglin, what roles they play in the development of HHT, and what this implies for future treatment of HHT patients. However, readers of this paper should keep in mind that the ligands for ALK1 and endoglin, and the pathways defective in HHT remain under debate.

## RECEPTORS AND SIGNALING PATHWAY INVOLVED IN HHT

The majority of HHT patients (80–85%) have been found to have mutations in *ACVLR1* or *ENG*.

ALK1 is a single transmembrane protein that comprises a cysteine-rich N-terminus extracellular ligand-binding domain [amino acids (aa) 22–118 encoded by exons 2, 3, and part of exon 4], a short transmembrane domain (119–141 aa encoded by exons 4, and part of exon 5), and a large intracellular domain (142–503 aa encoded by part of exon 5 up to exon10). The latter one includes the GS domain (a region rich in glycine and serine that is phosphorylated by a type II receptor), a serine/threonine kinase domain and a short cytoplasmic tail. Mutations in *ACVRL1* (375 entries to date) linked with HHT2 affect the integrity of each of these domains (http://www.arup.utah.edu/database/hht/) and 46% are missense variants.

Endoglin is an integral membrane glycoprotein composed of a 180-kDa disulfide-linked homodimer with a large and highly glycosylated extracellular domain presenting a multimodular structure (Gougos and Letarte, 1990; Bellon et al., 1993; Llorca et al., 2007; Gregory et al., 2014). Its extracellular domain is composed of an orphan domain (aa 26–359 encoded by exons 2–8), which does not share any homology with other proteins and has been described as the ligand-binding domain (Castonguay et al., 2011; Alt et al., 2012), and two zona pellucida domains (ZP-N aa 360–457 encoded by exons 9–11 and ZP-C aa 458–586 encoded by exons 12–14). The intracellular domain, 46 aa, is very short (aa 612–658 encoded by exon 15). Mutations in *ENG* (470 entries to date) linked with HHT1 also affect the integrity of each of these domains but only 21% are missense variants. The majority corresponds to deletion and splice defects leading to the expression of a truncated protein that is either not expressed at all or retained intracellularly, supporting a model of haploinsufficiency for HHT1 (Pece et al., 1997; Pece-Barbara et al., 1999; Cymerman et al., 2000; Paquet et al., 2001). However, it was also shown that some missense mutations that could not properly reach the cell surface could heterodimerize with wild-type (WT) endoglin and reduce the amount of endogenous WT endoglin at the plasma membrane. They could thus behave as dominant negatives, which could lead to more severe disease (Lux et al., 2000; Forg et al., 2014; Mallet et al., 2014).

The third gene involved in HHT encodes Smad4, the key downstream effector of TGFβ/BMP family signaling pathway. The *SMAD4* gene accounts for 2–3% of HHT cases and is associated with juvenile polyposis in most cases (Gallione et al., 2004, 2006, 2010).

Taken together these data support that HHT pathogenesis involves the ALK1/endoglin/Smad signaling pathway. Therefore, a better understanding of the physiological ligands of this receptor complex is required for more comprehensive knowledge of HHT pathogenesis.

## BMP9 AND BMP10 IN HHT

### EXPRESSION AND REGULATION OF BMP9 AND BMP10 ACTIVITIES

**FIGURE 2:**
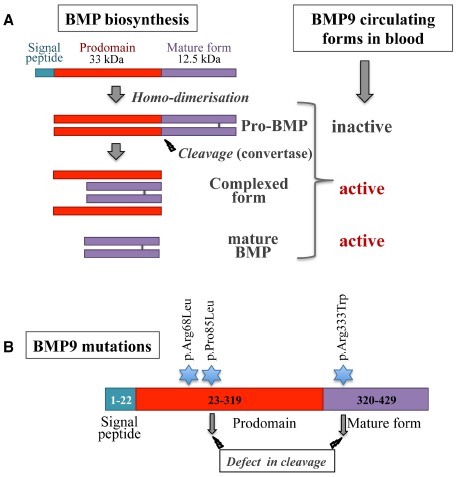
**(A)** Schematic diagram of BMP biosynthesis and processing. BMPs are synthesized as pre-pro-proteins that are disulfide-bonded to form pro-BMPs. Pro-BMPs represent a precursor inactive form that needs to be further processed. After cleavage by pro–protein convertases, the prodomains remain non-covalently associated with the mature BMPs. BMP9 complexed form has been shown to be able to activate ALK1 signaling (modified from Bidart et al., 2012). **(B)** BMP9 mutations found in HHT-like vascular syndrome. Three mutations have been described, indicated by a blue star. Two of these mutations are associated in vitro with a defect in BMP9 processing (modified from Wooderchak-Donahue et al., 2013).

BMP9 and BMP10 are two close members of the BMP family with 65% sequence identity at the protein level. They are both synthesized as a large precursor (Figure 2A) consisting of a prodomain for proper folding (aa 23–319 for BMP9, aa 22–316 for BMP10) and a C-terminal mature peptide (aa 320–429 for BMP9, aa 317–424 for BMP10) linked by a disulfide bridge within the mature domain. This protein is cleaved by subtilisin-related pro-protein convertases (e.g., furin; Susan-Resiga et al., 2011; Bidart et al., 2012) and following cleavage, the prodomain remains non-covalently associated with the mature dimer. At least for BMP9, this complex has been described as being able to bind to its receptor ALK1 and to activate its signaling pathway (Brown et al., 2005; Bidart et al., 2012; Figure 2A). In contrast, the BMP10 prodomain has been shown to confer latency to BMP10 on C2C12 cell activation (Sengle et al., 2011). Therefore, a major difference between these two BMPs is that only circulating BMP9 is biologically active; indeed, human plasma ALK1 activity using a BMP responsive reporter assay can be mostly inhibited by a neutralizing anti-BMP9 antibody, and murine plasma from *Bmp9*-knockout mice is not able to activate a BMP response (David et al., 2007; Ricard et al., 2012). However, we cannot exclude that there could be some active circulating BMP10, as shown by Chen et al. (2013).

BMP9 is mainly produced by the liver (Miller et al., 2000), more specifically by hepatocytes, and at a lower level by the lungs and the brain (Miller et al., 2000; Bidart et al., 2012). On the other hand, BMP10 is mainly produced by the heart, but also at a lower level by the liver and the lungs (Neuhaus et al., 1999). These two BMPs are present in both human and mouse plasma (David et al., 2008; Bidart et al., 2012; Ricard et al., 2012; Chen et al., 2013). Circulating levels of BMP9 and BMP10 have been shown to be around 0.5–15 ng/ml, higher than the EC_50_ described for binding to ALK1 (50 pg/ml; David et al., 2007). Therefore their circulating levels are sufficient to stimulate ALK1 and endoglin expressed on the endothelium. Although circulating BMP10 is inactive, recent data support a role for BMP10 on vascular development (Ricard et al., 2012; Chen et al., 2013). Thus further work is needed to elucidate BMP10 circulating forms and activation.

### BMP9 AND BMP10 BIND TO ALK1 AND ENDOGLIN

Both BMP9 and BMP10 have been shown to bind to ALK1 with a very high affinity (David et al., 2007; Scharpfenecker et al., 2007). For example, the Kd values for ALK1 extracellular domain (ALK1_ECD_) and BMP9 and for ALK3_ECD_ and BMP2 were respectively 29 and 330 nM (Mahlawat et al., 2012). The crystal structure of the ternary complex of recombinant mature BMP9 with the extracellular domains of ALK1 and ActR2B has revealed a novel orientation of ALK1 with respect to BMP9, which could explain the high affinity of BMP9 for ALK1 (Mahlawat et al., 2012; Townson et al., 2012). The majority of HHT2 mutations affect residues that are integral to the core structure and only three described HHT mutations (H66P, G79R, H87D) have been predicted to have a direct effect on BMP9 binding (Townson et al., 2012).

Interestingly, BMP9 and BMP10 are potentially the only TGFβ/BMP ligands that can bind to both type I and type II receptors with high affinity. This suggests a unique non-discriminative binding mechanism to generate the ternary complex ligand/receptor I/receptor II (Townson et al., 2012). In addition, although results differ from one study to another, the affinities of BMP9 and BMP10 for their type II receptors might be slightly different (Brown et al., 2005; Townson et al., 2012). Thus despite apparent sequence homology and possibly similar functions, BMP9 and BMP10 could act through different type II receptors.

Endoglin is a co-receptor, and so far it has not been shown to transduce a signal by itself. It has been demonstrated that it increases BMP9/BMP10/ALK1 signaling; however, the molecular mechanism of this effect is not understood yet (David et al., 2007; Scharpfenecker et al., 2007; Nolan-Stevaux et al., 2012). Endoglin has also been identified as a co-receptor for other members of the TGFβ family (Barbara et al., 1999). However, only BMP9 and BMP10 were shown to bind directly to endoglin (Castonguay et al., 2011; Alt et al., 2012; Gregory et al., 2014).

Binding studies have shown that endoglin and ALK1 do not compete for BMP9 binding, while endoglin and ActR2B competitively bind BMP9 (Castonguay et al., 2011). This is reflected in a model where endoglin and ALK1 act together to bind and capture BMP9 on the cell surface. The type II receptors then function to displace the bound endoglin to form a type I/type II receptor signaling complex.

### BMP9 AND BMP10 ROLES IN VASCULAR REMODELING

#### Evidence from in vitro studies

Many studies have attempted to elucidate the downstream cellular effects mediated by BMP9/BMP10/ALK1 signaling *in vitro* in endothelial cells, but no clear consensus has been reached. It has been proposed that pro- or anti-angiogenic roles for BMP9/BMP10 depend on the experimental system considered and the cell type used (David et al., 2007; Scharpfenecker et al., 2007; Upton and Morrell, 2009; Suzuki et al., 2010; Park et al., 2012). Taken together, the data do not allow us to propose a clear cellular mechanism for the BMP9/BMP10/ALK1 signaling pathway and further work will be necessary to integrate this pathway with other important signaling pathways in vascular development such as the VEGF (vascular endothelial growth factor) pathway. Our working model is that BMP9 and BMP10, present at significant levels in blood, may act via ALK1 as suppressors of endothelial cell migration and proliferation, maintaining a quiescent endothelial state (David et al., 2009; Atri et al., 2013).

#### Evidence from animal models: mice and zebrafish

*Bmp9* inactivation leads to viable mice with no obvious observable defect in blood vessels. However, it was found that these mice presented defects in lymphatic vessel maturation and valve formation, and lymph drainage deficiency, consistent with ALK1 expression in lymphatic endothelial cells (Levet et al., 2013; Yoshimatsu et al., 2013). In accordance with this result, *Alk1* deletion in mice has been described to lead to enlarged lymphatic vessels (Yoshimatsu et al., 2013). However, to our knowledge no lymphatic defects have been described so far in HHT patients.

On the other hand, *Bmp9* inactivation on its own did not lead to any defect in the retinal vascularization model (Ricard et al., 2012). However, injection of a neutralizing anti-BMP10 antibody in *Bmp9*-deficient mice strongly inhibited vascular expansion of the retina and induced an increase in vessel density, demonstrating a redundancy between BMP9 and BMP10 in vascular development (Ricard et al., 2012; Chen et al., 2013).

In contrast to *Bmp9*-deficient mice, *Bmp10*-deficient mice die very early, between E9.5 and E10.5, with profound defects in cardiac development (Chen et al., 2004), consistent with a high expression of BMP10 in the developing heart (Neuhaus et al., 1999) and BMP10 receptor ALK3 expression in cardiomyocytes (Briggs et al., 2013). However, very recently, the vascular status of these mice was reevaluated and it was found that *Bmp10*-deficient mice also presented very early vascular defects in the yolk sac and embryos (Chen et al., 2013). Moreover, these embryos developed arteriovenous malformations (AVMs) with a phenotype reminiscent of the vascular phenotype resulting from the loss of *Alk1* (Urness et al., 2000), indicating that BMP10 is a key ALK1 ligand during embryonic development. Taken together, these data suggest that BMP10 via ALK3 is a key player in cardiac development and that BMP10 via ALK1 plays a critical role in vascular development.

Similar observations could be made using another animal model, i.e., zebrafish. It was shown that embryos harboring a mutation in *Alk1* (mutant *violet beauregarde*, *vbg*) exhibit abnormal circulation albeit normal vessel patterning, with enlarged vessels resulting in robust cranial AVMs (Roman et al., 2002; Corti et al., 2011). Blocking Bmp9 expression by injecting *Bmp9* morpholinos led to mild impaired venous remodeling, suggesting that other ligands such as BMP10 might compensate for BMP9 (Wooderchak-Donahue et al., 2013). In this respect, *bmp10* morphants presented a phenotype that was indistinguishable from *Alk1* morphants (Laux et al., 2013): arteries were enlarged, contained supernumerary endothelial cells and AVMs. These data suggested that BMP10 is the important ligand for ALK1 in zebrafish in very early embryonic stages of vascular development.

Therefore, the current working model is that BMP10, which is expressed first, plays a key role during early embryonic development and could control endothelial cell numbers in nascent arteries (Chen et al., 2013; Laux et al., 2013). Then, as soon as BMP9 is produced, they are both involved in vascular development in an interchangeable manner and ensure vascular quiescence.

#### Evidence from HHT

The identification of BMP9 as a high affinity ligand for the receptor ALK1 and the co-receptor endoglin has resulted in the development of functional assays to study how a mutation affects the function of the mutated protein (Ricard et al., 2010; Mallet et al., 2014). These functional tests are important because they can discriminate a pathogenic mutation from a non-pathogenic mutation in the case of missense mutations. This is particularly relevant for patients with conflicting mutations (carrying, for example, two different mutations in *ENG* or *ACVRL1*) or a novel mutation.

In the case of *ENG*, two new missense mutations within the orphan domain (S278P, F282V) were recently described as inhibiting BMP9 binding to endoglin (Mallet et al., 2014) in accordance with the previous mapping of the BMP9-binding region of endoglin to its orphan domain (Castonguay et al., 2011; Alt et al., 2012) further supporting the involvement of BMP9 in HHT pathogenesis.

Recently, *GDF2* (encoding BMP9) mutations have been described in a vascular anomaly syndrome with phenotypic overlap with HHT (Wooderchak-Donahue et al., 2013). Mutations were identified in three unrelated probands (pArg68Leu, pPro85Leu, and pArg333Trp), the first two being within the prodomain and the last one in the mature peptide (Figure 2B). The last two BMP9 mutations were shown to affect BMP9 processing, but further work is needed to show how these mutations affect the circulating levels of BMP9 or its signaling to ALK1 or endoglin.

According to what has been observed, *in vitro* in endothelial cells and *in vivo* in mice and zebrafish, HHT could result from a defect in the angiogenic balance. In accordance with this hypothesis, several case reports and clinical trials using antibodies against VEGF (bevacizumab from Roche/Genentech) have been published in which HHT patients were successfully treated (Flieger et al., 2006; Mitchell et al., 2008; Dupuis-Girod et al., 2012; Lupu et al., 2013; Vlachou et al., 2013).

## CONCLUSION AND PERSPECTIVES

Taken together, recent data clearly indicate that the BMP9/BMP10/ALK1/endoglin pathway is an emerging new signaling pathway that is critical for vascular development and defective in the vascular pathogenesis of HHT. Although not discussed here, this pathway is also involved in pulmonary arterial hypertension (PAH), another rare vascular disease associated with mutations in *BMPR2*, *ACVRL1*, and *ENDOGLIN* (Trembath et al., 2001; Girerd et al., 2010). Our current working model is that BMP9 and BMP10 are present in blood, bind to ALK1 and endoglin on endothelial cells, and induce vascular quiescence. When ALK1 or endoglin is mutated, signaling through this pathway is decreased and may lead to an increase in the angiogenic response. This model is supported by the beneficial treatment of HHT patients with anti-angiogenic drugs (bevacizumab; Dupuis-Girod et al., 2012). Other therapeutic approaches for HHT would be to stimulate this deficient pathway, either via drugs that activate the pathway as has been recently described for PAH (Spiekerkoetter et al., 2013) or by adding more BMP9 or BMP10 ligands or using mimicking peptides. Indeed, although high levels of BMP9 and BMP10 are present in blood they might not be under circulating bioavailable forms. The identification of the ligands involved in this signaling pathway represents real progress in the understanding of the pathogenesis of HHT and should lead to new therapeutic developments in the future.

### Conflict of Interest Statement

The authors declare that the research was conducted in the absence of any commercial or financial relationships that could be construed as a potential conflict of interest.
